# Comparison of mechanical properties of beta-titanium wires between leveled and unleveled brackets: an *in vitro* study

**DOI:** 10.1186/s40510-014-0042-0

**Published:** 2014-05-30

**Authors:** Natalia Martins Insabralde, Thaís Poletti, Ana Cláudia Conti, Paula Vanessa Oltramari-Navarro, Murilo B Lopes, Carlos Flores-Mir, Marcio Rodrigues de Almeida

**Affiliations:** Department of Orthodontics, University of North Parana, UNOPAR, Londrina, Paraná Brazil; Department of Restorative Dentistry and Biomaterials, University of North Parana, Londrina, PR Brazil; Division of Orthodontics, University of Alberta, Edmonton, Alberta Canada

**Keywords:** Orthodontic wires, Beta-titanium, Three-point bending test

## Abstract

**Background:**

The objective of this study is to evaluate the force-deflection behavior of beta-titanium alloy wires between two leveled and unleveled bracket alignment scenarios using a three-point bending test.

**Methods:**

Six groups of ten beta-titanium alloy wire segments (0.017 × 0.025-in. diameter) of different manufacturers (Orthometric, Ortho Organizers, GAC, Morelli, and Ormco) were used. Both brackets were bonded to an acrylic jig with a 10-mm interbracket distance. A 1-mm deflection test in two hypothetical conditions (with aligned brackets and by simulating a 2-mm horizontal displacement of the brackets) was explored. Forces of activation and deactivation of the wires during both tests were compared by an analysis of variance (ANOVA) tests followed by a Tukey test.

**Results:**

A statistically significant difference was found in the force-deflection behavior between some of the wires in both simulated *in vitro* conditions. For the leveled-type alignment scenario, the differences between wires were up to 70 g (range 110 to 179 g). For the unleveled-type alignment scenario, these differences were up to 65 g (range 111 to 175 g).

**Conclusions:**

The study showed some significant differences in forces generated during activation and deactivation among the five types of beta-titanium wires tested. In comparing leveled and unleveled brackets during activation, only Orthometric Beta Flexy and Ormco Beta-titanium were different between them.

## Background

Nowadays, orthodontists can select from among several available wire alloys the ones that better meet their specific demands on any given clinical situation. Thus, to be familiar with the mechanical properties and the clinical applications of those wire alloys is indispensable [1]. In fact, knowledge on the mechanics of an orthodontic system is essential in order to reach orthodontic results that are both desirable and predictable [2].

Beta-titanium (β-Ti) alloy wires were first introduced in Orthodontics in 1979 [2,3]. These wires have gained great popularity over the past few years, due to their unique combination of properties (biocompatibility, resistance to corrosion, and low stiffness) [4]. Today, they are highly sought to make intrusion arches, cantilevers, and closing loops, by facilitating an individualized dental movement through controlled force systems [5]. The correct utilization of beta-titanium alloy wires can lead to a more efficient orthodontic tooth movement over a shorter period of time [6]. They may also be clinically adequate during the alignment and leveling of teeth throughout the orthodontic treatment [7]. The beta-titanium alloy wires provide an adequate combination of spring back, average stiffness, and good formability, therefore, providing a smaller permanent deformity of these wires [1]. Some authors concluded that an average low deflection force is beneficial for the initial phase of an orthodontic treatment, since it offers light and more constant forces and better precision in force application [8,9].

A questionnaire about the action and applicability of beta-titanium alloys in clinical practice has been completed. It was found that the use of beta-titanium alloys is still growing as compared to stainless steel alloys, especially during the initial stages of treatment. While their use was of 13.5% in 2002, in 2008, it grew to 15.9%. During the final stage of treatment, the use of beta-titanium alloys was of 16.6% in 2002 and increased to 23.9% in 2008. When introduced, these wires were used in loops and cantilevers within the segmented arch technique. Today, they are becoming popular in all phases of orthodontic treatment [10].

The modulus of elasticity of a wire describes the resistance of the material to flexion. It is considered the most important clinical parameter because it closely affects the biological nature of dental movement [11]. Various studies [2-4,11,12] have evaluated orthodontic wires in laboratorial *in vitro* tests during deflection tests, in order to assess their load/deflection behavior and their elasticity module. All these studies used a set up where the brackets where dimensionally leveled and aligned. In an attempt to better simulate a different clinical situation, a 2-mm horizontal step between the brackets will be tested to evaluate if the load/deflection behavior changes. The objective of the study was therefore to evaluate the behavior of the deflection force, modulus of elasticity, and plastic deformation of five commercially available beta-titanium alloy wires between two leveled and unleveled bracket alignment scenarios using the three-point bending test.

## Methods

The present study followed the ISO 15.841 guideline to perform orthodontics tests. As for the force-deflection tests, the ISO norms indicate the three-point bending test as the most appropriate.

**Figure 1:**
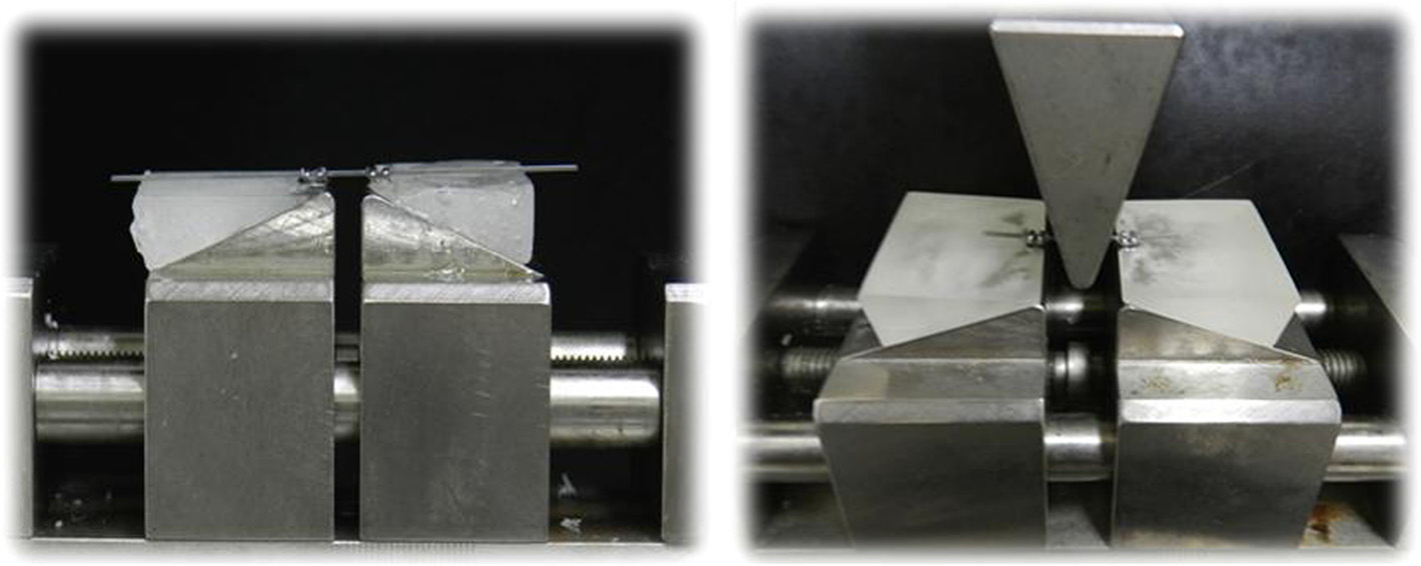
**Three-point bending test showing the deflection of the wire with aligned brackets.**

**Figure 2:**
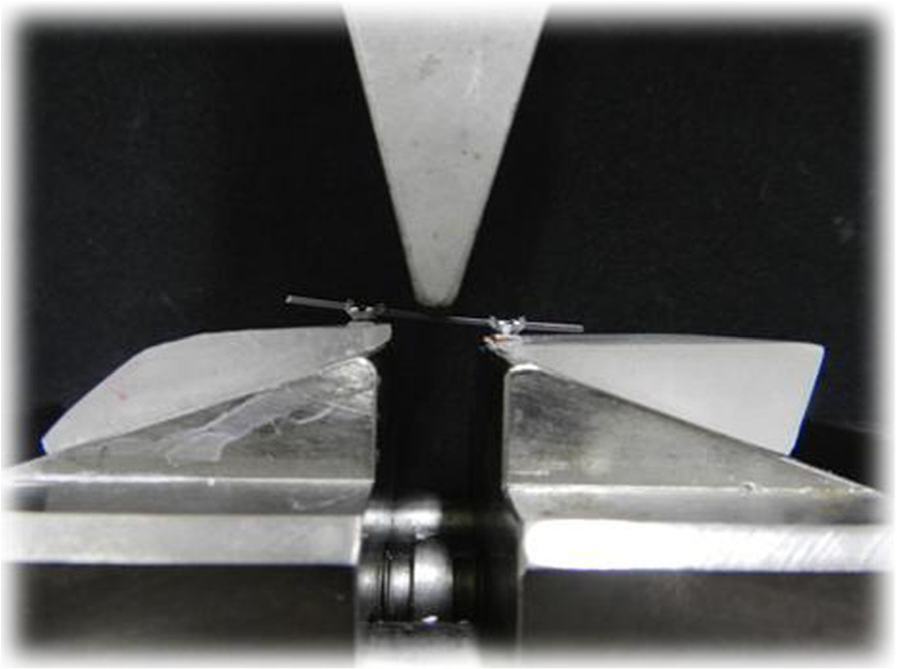
**Load-deflection apparatus with the specimen under load in the 2-** **mm horizontal displacement brackets.**

The deflection test utilized a device fabricated to allow one-point deflection using two brackets (interbracket distance of 10 mm) (Figure 1). The device had a 10-mm-diameter acrylic rod adapted to a metallic frame. During the tests, a specially designed jig made of acrylic was bonded onto the machine's support. Edgewise brackets (0.018 × 0.025-in. slot) without angulation or torque (Twin mini, Morelli, Sorocaba, Brazil) were bonded to the acrylic jig. A segment of 0.017 × 0.025-in. wire was used to set the two brackets parallel for the first part of the experiments. In order to simulate a clinical situation of misaligned teeth, during the second part of the experiment, a 2-mm horizontal displacement of the brackets was incorporated (Figure 2).

The test wire specimens were fixed on the bracket with elastomeric ligature (Morelli, Sorocaba, Brazil). Six different 0.017 × 0.025-in. beta-titanium alloy wires, 30 mm in length and marketed by five companies, were tested (Table 1). With regard to the Orthometric brand, we analyzed two types of wires (the Orthometric Beta Flexy which is composed of a singular beta-titanium alloy wire and the Orthometric Flexy Multi which combines a beta-titanium alloy wire with a nickel-titanium one).

**Table 1:** **Manufacturers, commercial names, and codes of beta-titanium wires tested**

**Manufacturer**	**Commercial name**	**Code/material (** ***N*** **)**	**Lot number**
GAC, Islip, NY, USA	Resolve	TMA (10)	F1013282
Morelli, Sorocaba, SP Brazil	Beta III	TiMo (10)	1519074
Ormco, Glendora, CA, USA	TMA	TMA (10)	8F106F
Orthometric, Marília, SP, Brazil	Flexy Multi	TMA-NiTi (10)	1109
Orthometric, Marília, SP, Brazil	Beta Flexy	TMA (10)	000149
Ortho Organizers, Carlsbad, CA, USA	CNA Beta	Beta CNA (10)	F1018428

All the groups were tested at a controlled temperature of 25°C, SD ±1.

The same investigator cut all wire segments with the aid of a digital caliper (Mitutoyo, Japan). ISO Norm 15.841 advocates a wire sample of at least six specimens, in order to possess a greater certainty in the results observed. Ten specimens of each wire [4] were placed individually in the bracket slot and ligated with a ligature wire. After securing each wire specimen, the acrylic rod was attached to the support utilized for the deflection test. Loading was achieved through movement of a metal loading device adapted on a universal mechanical testing machine (Emic, DL 2000, São José dos Pinhais, Brazil) with a 5-kg load cell and a crosshead speed of 0.5 mm/min [12].

A total of 60 segments of 0.017 × 0.025-in. β-Ti wires were tested. The midportion of the test wire specimen was deflected. Forces of the deflection tests were measured in intervals as follows: 0.5, 1, 1.5, and 2 mm. The evaluations of the load-deflection of the wires were considered the unloading forces. Forces necessary for the deformation test were recorded directly into the computer using a Tesc version 3.04 software, Copyright ^c.^ 1998-2005 Mattest Automação e Informática Ltda.

An activation deflection of 1 mm was selected to establish a comparison parameter [4,6,13-15] at the midpoint of the loading deflection used in this study. According to the ISO 15.841 standard, the test deflection of the wire should be evaluated from 0.5 to 0.5 mm.

### Statistical analysis

Means and standard deviations of the forces generated during the unloading by the 1-mm deflection were selected for the statistical comparison of the data [6,10,13]. In order to verify a normal distribution of the data, a Kolmogorov-Smirnov test was used. The results of this test demonstrated that data showed a normal distribution. Therefore, data were analyzed through the parametric analysis of variance (ANOVA) test followed by Tukey test. In all tests, a 5% level of significance was adopted (*p* < 0.05). The statistical procedures were carried out in the software Statistica version 12 (StatSoft Inc., Tulsa, USA).

## Results

Means and standard deviations of the modulus of elasticity during the activation of the three-point bending test are listed in Table 2. The modulus of elasticity during the activation of the three-point bending test showed a statistically significant difference between the brands. One that presented the highest value was Morelli (45.58 GPa), showing a higher wire stiffness wire, and the lowest value was GAC (32.98 GPa) along with Orthometric Flexy Multi (33.01 GPa) and Ortho Organizers (34.16 GPa) brands. These brands showed a lower wire stiffness.

**Table 2:** **Means and standard deviations of modulus of elasticity during the activation of three-point bending test**

**Commercial brand**	**Three-point bending test (GPa)**	
	**Mean**	**SD**
GAC	32.98 a	1.13
Morelli	45.58 c	9.45
Ortho Organizers	34.16 ab	2.15
Ormco	37.89 bc	7.12
Orthometric (Beta Flexy)	41.33 c	3.22
Orthometric (Flexy Multi)	33.01 a	5.82
*P* value	<0.001*	

*Statistically significant differences (*p* < 0.05). Similar letters represent non-significant differences.

Means and standard deviations of the 1-mm deflection activation force between leveled and unleveled brackets can be seen in Table 3. The ANOVA test showed a difference between the manufacturers of beta-titanium wires when comparing between the brands. These differences in the brackets leveled were up to 70 g from a range between 110 g (Orthometric Flexi Multi) to 179 g (Morelli). During the activation of the wires, the lowest released force at the 1-mm deflection occurred for the brand Orthometric Flexy Multi. The Ormco, Ortho Organizers, and GAC did not differ from each other (range between 139 and 147 g). On the other hand, the brands Morelli and Orthometric Beta Flexy showed the greatest forces in load-deflection behavior, but they did differ from each other. However, in unleveled bracket results, the lowest released force was the Orthometric Flexy Multi brand that differed from Ormco. The latter did not present any differences from the brands Ortho Organizers, GAC, and Orthometric Beta Flexy (range between 139 and 142 g). Morelli showed the greatest force in load-deflection behavior (175 g). Comparing the scenarios, only Orthometric Flexy Multi and Ormco brands showed a statistically significant difference among the groups.

**Table 3:** **Comparison of load/deflection behavior between leveled and unleveled brackets during a 1-mm deflection in activation evaluation**

**Commercial brand**		**Activation**	
		**Mean (g)**	**SD**
Leveled	Ortho Organizers	142.40 b	2.71
	Morelli	179.47 d	3.02
	GAC	147.15 b	2.06
	Orthometric (Beta Flexy)	160.39* c	11.81
	Orthometric (Flexy Multi)	110.25 a	6.03
	Ormco	139.62* b	6.18
Unleveled	Ortho Organizers	140.15 c	1.16
	Morelli	175.68 d	5.10
	GAC	139.38 c	3.80
	Orthometric (Beta Flexy)	142.44* c	13.27
	Orthometric (Flexy Multi)	111.05 a	8.60
	Ormco	128.84* b	7.22

*Statistically significant differences (*p* < 0.05). Different lowercase letters represent non-significant differences.

The values obtained by ANOVA test in the comparison of the load/deflection behavior between leveled brackets and unleveled brackets during a 1-mm deflection in deactivation are shown in Table 4. For the leveled-type scenario, these differences were also up to 60 g from a range between 3 to 64 g. The highest force release during the deactivation was the Ormco (64 g) that did not differ from GAC and Ortho Organizers. This shows that the two aforementioned wires showed the best spring back behavior. On the other hand, brands Morelli and Orthometric Beta Flexy showed the lowest force during the deactivation. For the unleveled-type scenario, they showed the same amount of force between the brands. In the comparison of scenarios, only Morelli brand showed statistically significant difference between the groups.

**Table 4:** **Comparison of load/deflection behavior between leveled and unleveled brackets during a 1-mm deflection in deactivation evaluation**

**Commercial brand**		**Deactivation**	
		**Mean (g)**	**SD**
Leveled	Ortho Organizers	56.65 c	1.82
	Morelli	3.18* a	0.61
	GAC	57.99 cd	1.21
	Orthometric (beta Flexy)	3.54 a	1.26
	Orthometric (Flexy Multi)	37.32 b	6.42
	Ormco	64.08 d	1.75
Unleveled	Ortho Organizers	59.10 c	0.69
	Morelli	13.71* a	1.05
	GAC	62.06 cd	1.43
	Orthometric (beta Flexy)	9.61 a	4.31
	Orthometric (Flexy Multi)	34.09 b	1.45
	Ormco	68.29 d	2.61

*Statistically significant differences (*p* < 0.05). Different lowercase letters represent non-significant differences.

Table 5 shows the mean and standard deviation when comparing brands and scenarios for the plastic deformation. In the leveled brackets, the brands Ormco, Ortho Organizers, GAC, and Orthometric Flexy Multi showed the best values of plastic deformation and did not show statistically significant difference among them. However, Morelli and Orthometric Beta Flexy demonstrated the worst values among the brands without differences between each other. The results obtained in unleveled brackets showed that Ormco and GAC have the best results; however, they did not differ from the Ortho Organizers and Orthometric Flexy Multi, which also did not differ from Morelli. The latter did not differ from Orthometric Beta Flexy. Only Orthometric Flexy Multi did not show statistically significant difference between the two scenarios.

**Table 5:** **Mean and standard deviation values (in mm) in leveled and unleveled bracket scenarios for plastic deformation**

**Commercial brand**		**Plastic deformation (mm)**	
		**Mean**	**SD**
Leveled	GAC	0.53*	0.00
	Morelli	0.97*	0.00
	Organizers	0.53*	0.00
	Ormco	0.50*	0.02
	Orthometric (beta Flexy)	0.98*	0.02
	Orthometric (Flexy Multi)	0.55	0.08
Unleveled	GAC	0.43*	0.02
	Morelli	0.90*	0.00
	Organizers	0.46*	0.00
	Ormco	0.36*	0.02
	Orthometric (beta Flexy)	0.92*	0.03
	Orthometric (Flexy Multi)	0.53	0.12

*Statistically significant differences (*p* < 0.05) for the same wire.

## Discussion

It is well known that an archwire for any given clinical situation is selected taking into account the mechanical properties of the alloy. Ideal archwires should possess a good balance of stability, stiffness, resilience, and formability [2]. Beta-titanium alloy wires have been widely used in orthodontic practice because of their favorable characteristics such as low stiffness, excellent formability, and efficiency in tooth movement. The results of the present study support the stated kinder nature of beta-titanium archwires when compared to similar values reported for stainless steel alloys [3,11,15,16].

In this study, a three-point bending test was performed to evaluate the load-deflection property, which is one of the most important parameters in determining the biologic response of tooth movement [13,17]. Clinicians appeared to be concerned with knowing what force is produced by the wire in relation to the amount of deflection [13,18].

This *in vitro* test was conceived to simulate a deflection that should induce dental movement. The beta-titanium alloy wires showed varied values of modulus of elasticity during activation. Some brands certainly showed a greater modulus of elasticity than others. GAC, Ortho Organizers, Ormco, and Orthometric Flexy Multi showed the lowest values of the modulus of elasticity in the three-point bending test, i.e., had the lowest stiffness of the beta-titanium wires. These brands, therefore, may be required in clinical situations that need loops, such as T-loop or mushroom loop archwires [19,20]. Our study showed that clinically, one can assume that the lower the stiffness, the better the TMA wire (Ormco, Glendora, CA, USA) and also the better the spring back effect.

The tested beta-titanium alloy wires showed a statistically significant difference in load/deflection behavior and indicate that different forces exist for the same amount of deflection even though the ‘same’ alloy is used. Among the six types of archwires analyzed during an activation of 1 mm in both scenarios, the brands Orthometric Flexy Multi, Ormco, Ortho Organizers, and GAC showed the lowest values of force. Overall, all the brands tested showed an adequate force for dental movement [21]. At the same time, the archwires analyzed during the deactivation of 1 mm in both scenarios, Ormco, GAC, and Ortho Organizers, showed that they are the best wires for this behavior; they demonstrated the greatest forces. Further confirming this, the plastic deformation analysis showed the same tendencies, including those for the Orthometric Flexy Multi which showed the best results. Clinically, the wires that had the greatest values in deactivation are considered better because they can increase the duration time of tooth movement, reducing the final treatment time.

**Figure 3:**
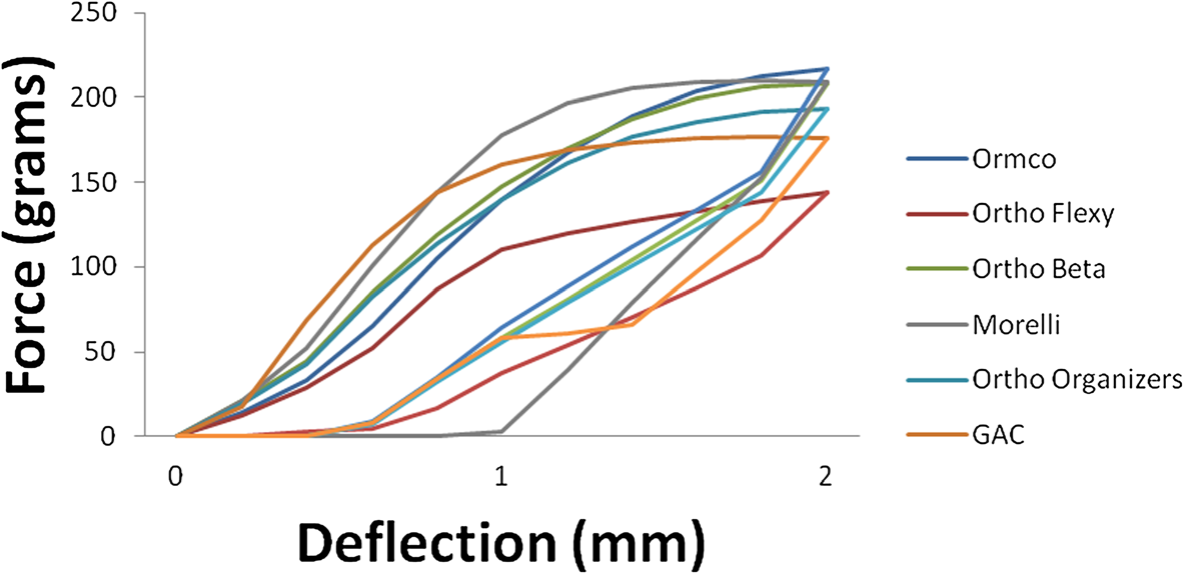
**Average force-deflection curves for beta-titanium wires with aligned brackets during activation and deactivation.**

The beta-titanium alloy wires showed plastic deformation during activation. The force-deflection curves distinguished wires that exhibited more plastic deformation than others (Figure 3). The brands Morelli and Orthometric Beta Flexy required greater force to deflect and had more plastic deformation at the end of the deactivation curve. Therefore, it is advisable that these beta-titanium alloy wires would be selected for clinical situations that require bends in the archwire, i.e., loop springs or cantilever.

All beta-titanium alloy wires tested exhibited statistical differences, indicating the existence of different forces for the same amount of deflection when using different commercially available wires. Among the six types of wires analyzed in both activation and deactivation phases, only Morelli and Orthometric Beta Flexy wires exhibited forces different from the others. These two brands exhibited the highest force on activation, indicating that Morelli and Orthometric Beta Flexy have higher stiffness during deflection. At a deactivation deflection of 1 mm, Morelli produced a force of 3.18 g. This force is not enough to induce the biological response needed to produce dental movement in most patients [21]. The Orthometric Flexy Multi, wire showed forces different from those of the other wires tested. Also, Ormco, Ortho Organizers, and GAC wires showed low forces with activation of 1 mm.

The flexibility and the load/deflection relationship depend on the modulus of elasticity [22]. The lower this relationship, the more constant is the force that makes a tooth move. With a variation of the modulus of elasticity of the wires, progressive stiffness of the archwires may be achieved without altering the cross section of the wires and have, as advantages, the use of rectangular wires in the first stages of treatment, guidance as to the choice or the wires, and less frequent changes [22].

**Figure 4:**
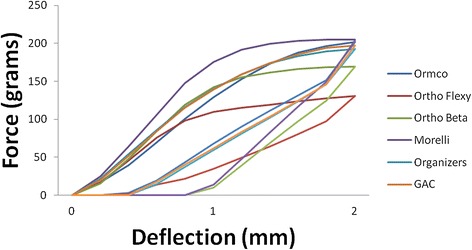
**Average force-deflection curves for beta-titanium wires with 2-** **mm displacement brackets during activation and deactivation.**

The representative bending plots for the force-deflection curves of wires with unleveled brackets (Figure 4) showed that only Morelli did not show a statistically significant difference between leveled and unleveled brackets for the deactivation. Regarding activation, only Orthometric Flexy Multi and Ormco did not show statistically significant difference. Moreover, great similarity between the curves displayed for both manufacturers can be seen. Also, only Orthometric Flexy Multi needed less force to deflect.

Undoubtedly, the wires that need lower forces to deflect represent a favorable characteristic for dental movement and can be successfully used in the control of the system of forces between tooth and periodental structures. It is worth mentioning that future studies with different displaced bracket distances and directions are required to complement the results of this study. The clinical importance of such differences remains unclear, but an argument can be made that almost 100 g (lowest against highest force values) difference should no be easily clinically dismissed.

### Limitations

Laboratory (*in vitro*) tests do not necessarily reflect clinical situations, but these tests provide a basis for comparison of different wires and have been used in many studies in the literature. These tests may be used as stepping stones to better justify more expensive clinical studies. The results from such *in vitro* tests should be further tested whenever possible in clinical trials [23].

Orthodontic tooth movement occurs when a force is exerted on teeth. In this study, we assessed a deflection force of 2 mm and results obtained from this degree of displacement. The brackets were unleveled to produce a clinical situation where there is presence of unleveled teeth. In this situation, there is a larger friction in brackets by changing the force required for the displacement, therefore if the values differ from the values found in a situation of a classic three-point bending test. If this deflection force is larger than 2 mm, it may produce different forces and consequently also different results. The type of ligation may have an effect. Only ligature wires where used. Finally, the effects when more than two brackets are considered were not evaluated.

## Conclusions

The following conclusions are drawn from the study:

- The study showed significant differences in force during activation and deactivation among the five types of beta-titanium wires tested.
- In comparing leveled and unleveled brackets during activation, only Orthometric Beta Flexy and Ormco Beta-titanium wires show a statistically significant difference between them. In the deactivation part, only Morelli titanium alloy wire showed a statistically significant difference.
- Among the commercial brands studied, those that showed the best behavior in their capacity to maintain an elastic memory in the three-point bending test were as follows: Ormco, GAC, Ortho Organizers, and Orthometric Flexy Multi Beta-titanium wires.
- The behavior of the load-deflection relationship showed that Ormco, Ortho Organizers, and GAC Beta-titanium wires obtained the best force values during deactivation.
